# Impact of Upper Airway Characteristics on Disease Severity and CPAP Therapy in Chinese Patients With OSA: An Observational Retrospective Study

**DOI:** 10.3389/fneur.2022.767336

**Published:** 2022-03-04

**Authors:** Cheng Zhang, Mingxin Chen, Yane Shen, Yuhong Gong, Jing Ma, Guangfa Wang

**Affiliations:** ^1^Department of Respiratory and Critical Care Medicine, Peking University First Hospital, Beijing, China; ^2^Department of Respiratory and Critical Care Medicine, The First Affiliated Hospital of Harbin Medical University, Harbin, China

**Keywords:** obstructive sleep apnea, upper airway, awake endoscopy, collapse, CPAP compliance

## Abstract

**Objective:**

The characteristics of the upper airway (UA) are important for the evaluation and treatment of obstructive sleep apnea (OSA). This study aimed to investigate the association of UA characteristics with OSA severity, titration pressure, and initiation of and 3-month compliance with continuous positive airway pressure (CPAP).

**Methods:**

This retrospective study included consecutive patients examined using a semi-quantitative UA evaluation system (combination with physical examination and awake endoscopy) during 2008–2018 at the Department of Respiratory and Critical Care Medicine, Peking University First Hospital. First, the differences in UA characteristics were compared between patients with simple snorers and mild OSA and those with moderate-to-severe OSA. Then, the effect of UA characteristics on the initiation to CPAP therapy and 3-month adherence to CPAP was conducted.

**Results:**

Overall, 1,002 patients were included, including 276 simple snorers and patients in the mild OSA group [apnea-hypopnea index (AHI) <15] and 726 patients in the moderate-to-severe OSA group (AHI ≥15). Tongue base hypertrophy, tonsillar hypertrophy, mandibular recession, neck circumstance, and body mass index (BMI) were independent risk factors for moderate-to-severe OSA. Among those patients, 119 patients underwent CPAP titration in the sleep lab. The CPAP pressures in patients with thick and long uvulas, tonsillar hypertrophy, lateral pharyngeal wall stenosis, and tongue hypertrophy were higher than those of the control group (*P* < 0.05, respectively). The logistic regression analysis showed that nasal turbinate hypertrophy, mandibular retrusion, and positive Müller maneuver in the retropalate and retroglottal regions were independent predictors for the initiation of home CPAP treatment.

**Conclusion:**

Multisite narrowing and function collapse of the UA are important factors affecting OSA severity, CPAP titration pressure, and the initiation of home CPAP therapy. Clinical evaluation with awake endoscopy is a safe and effective way for the assessment of patients with OSA in internal medicine.

## Introduction

Obstructive sleep apnea (OSA) is a common sleep disorder characterized by repetitive complete (apneas lasting for >10 s) or partial (hypopnea) upper airway (UA) collapse leading to intermittent hypoxia (1, 2), with a prevalence of 6–13% in adults (3). It is associated with chronic and systemic inflammation, several biomarkers, and comorbidities such as neurovegetative disorders, cardiovascular, and nasal inflammation (4, 5). Although neuroregulation plays an important role in OSA, UA (nasal, glossopharyngeal, and oropharyngeal) features and collapse are also important influencing factors for OSA (6). Previous studies used imaging modalities, including radiography, CT (7), and MRI (6), for UA assessments. Yet, these imaging modalities can only perform static observations, not functional assessments. The use of these modalities is also limited by the associated radiation exposure and complex instrument design.

Transnasal endoscopy is a well-established method for visualizing and evaluating the UA (8). There are mainly three methods of endoscopy evaluation: awake electronic endoscopy, drug-induced sleep endoscopy (DISE), and natural sleep endoscopy. Drug-induced sleep laryngoscopy represents the gold standard for the diagnosis of obstructive site (9) and is often used for preoperative evaluation in ear, nose and throat (ENT) surgeries (10). However, it carries some risk due to the drugs, requires closer monitoring, and is more complex and expensive. Natural sleep endoscopy can observe the UA under normal sleep conditions, but it is time-consuming and laborious and requires a longer hospital visit, which is not conducive to routine outpatient examination (11, 12). Relatively, awake supine laryngoscopy is more convenient and safer for patients with OSA in an internal medicine department; it is a convenient visual modality to evaluate the UA, including its collapsibility, using the Müller maneuver (8).

Continuous positive airway pressure (CPAP) is the first-line treatment and the primary treatment in internal medicine-led sleep centers (2, 13). Therefore, sleep centers in internal medicine departments currently tend to pay less attention to the characteristics of the UA in patients with OSA. Evaluation of the UA is mainly performed in the surgical setting. However, understanding the anatomy of the UA, sites of obstruction, and collapse pattern is crucial to elucidate the pathogenesis of OSA and characterize its subtypes and may be associated with CPAP treatment. Some studies showed that CPAP titration levels were positively correlated with the UA length (UAL) and the mandibular plane to hyoid bone (*p* < 0.05) assessed using CT (14). Nasal and pharyngeal obstruction was significantly higher in the CPAP nonadherent group than in the CPAP adherent group (15). However, the relationship between the UA configuration and the efficacy of and adherence to CPAP remains unclear.

Therefore, this study aimed to investigate the UA characteristics in Chinese patients with OSA managed by the internal medicine team using awake evaluation and to investigate the association of the UA characteristics with OSA severity, influence on titration, and initiation of and 3-month adherence to CPAP.

## Methods

### Study Design and Patients

This cross-sectional study included consecutive patients referred to the authors' sleep laboratory with a chief complaint of snoring and who underwent awake endoscopy between 2008 and 2018 at the Department of Respiratory and Critical Care Medicine of Peking University First Hospital.

Inclusion criteria were: (1) >18 years of age and (2) polysomnography (PSG) study and awake UA evaluations performed at the department.

Exclusion criteria were failure to perform or complete an overnight PSG monitoring or awake laryngoscopy, resulting in incomplete data or age ≤18 years.

This study was approved by the Ethics Committee of Peking University (2020-562) and complied with the principles of the Declaration of Helsinki. The requirement for informed consent was waived considering the retrospective nature of this study.

**Figure 1:**
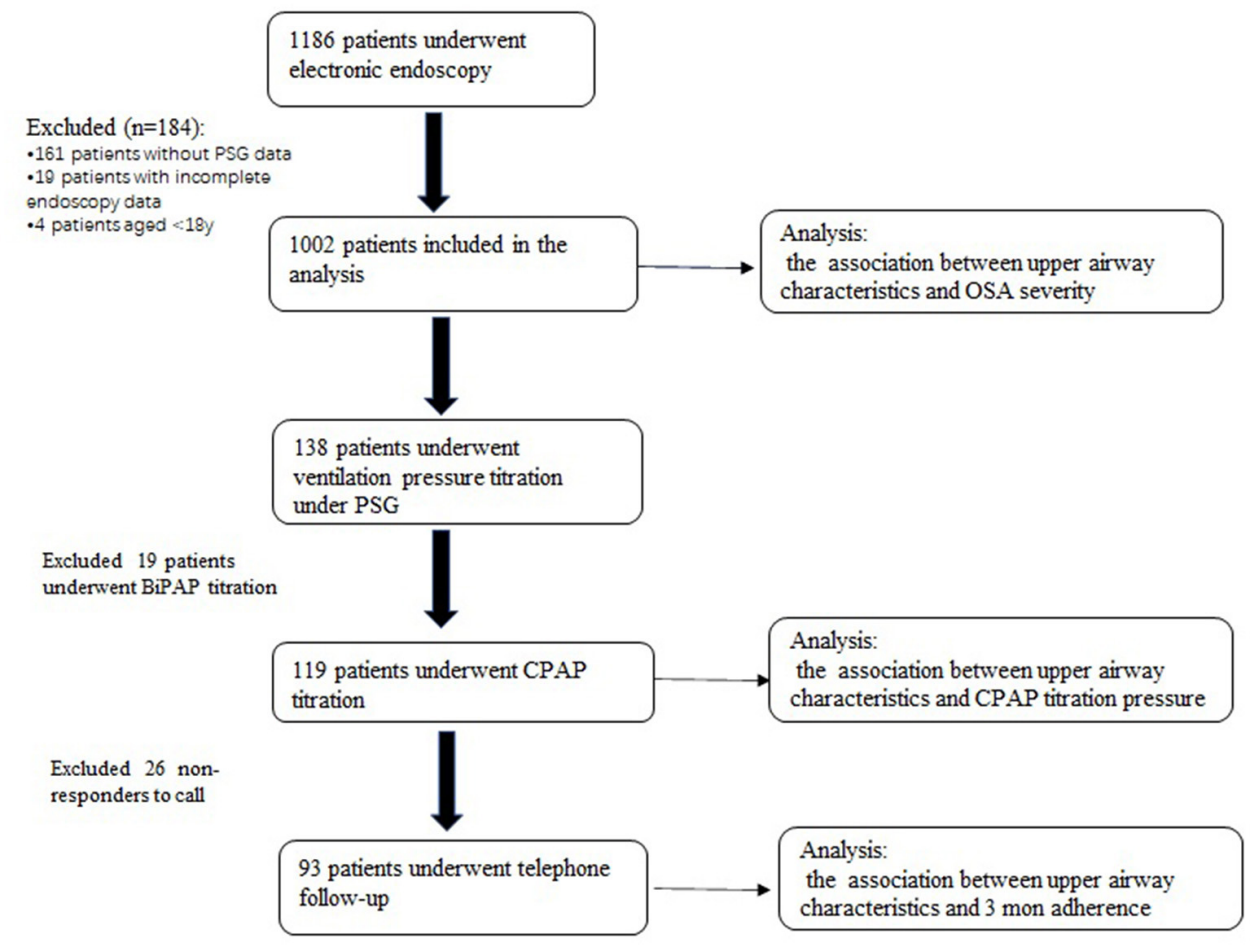
Patient enrollment flow chart.

The flowchart of the enrollment is shown in Figure 1.

## Data Collection And Definition

The semi-quantitative UA evaluation system established in a previous study by the authors' group (16) was combined with physical examination, endoscopy, and Müller maneuver examination. Patients were examined at the waking state.

Data of the height of the soft palate, length of the uvula, size of the bilateral tonsils, and condition of the lateral pharyngeal wall were collected. Data on the presence of nasal septum deviation, inferior turbinate hypertrophy, nasal polyps or other masses, mucosal edema, or secretions caused by rhinitis were also collected. Data of the collapsibility of the UA, collapse of the pharyngeal walls, and the base of the tongue were then collected (16–18).

The size of the tongue was recorded. Tonsils were classified according to their size as grades I, II, and III. Tonsil size is usually classified into grades 0–4 (19, 20). In this study, for simplicity, we classified the tonsil size into the following three grades: grade 1 (defined as tonsils absent or tonsils hidden inside the pillars or tonsils extending to the pillars), grade 2 (defined as tonsils extending beyond the pillars), and grade 3 (defined as tonsils extending to the midline). Grades 2 and above suggest tonsillar enlargement.

Examination of the tongue size can be performed with the tongue relaxed, using the occlusal plane of the mandibular teeth. If the tongue is at or below the level of the occlusal plane of the lower teeth, then the tongue is considered to be of normal size. If the tongue is beyond this level, then the tongue is considered to be enlarged (21).

The height of the oropharynx was evaluated using the Friedman palate position score (22, 23) based on visualization of structures in the mouth, with the mouth open widely without protrusion of the tongue. Grade I indicated visualization of the entire uvula and tonsils. Grade II indicated visualization of the uvula, but not the tonsils. Grade III allows visualization of the soft palate, but not the uvula. Grade IV allows visualization of the hard palate only. Grades III and IV generally represent a relatively low position of the soft palate.

The obstruction of the regions during Müller maneuver was measured according to a semi-quantitative method and it can be divided into four levels: no obstruction or up to 25, 50, 75, and 100% of obstruction (8). In this study, a positive result in Müller maneuver in the retropalate (RP) was defined as the collapse area being more than 75% and a positive result in Müller maneuver in the retroglottal (RG) was defined as the collapse area being more than 50%. Data on the pharyngeal space were collected using a four-point ordinal scale (24). Class I indicated that the palatopharyngeal arch intersected at the edge of the tongue. In class II, the palatopharyngeal arch intersected at ≥25% of the tongue diameter. In class III, the palatopharyngeal arch intersected at ≥50% of the tongue diameter. In class IV, the palatopharyngeal arch intersected at ≥75% of the tongue diameter. Classes III and IV indicated lateral pharyngeal wall narrowing.

The shape of the retropalatal region was classified as flat oval (i.e., the anterior and posterior diameters were less than the right and left diameters), long oval (i.e., the anterior and posterior diameters were greater than the right and left diameters), or quasicircular (i.e., the anterior and posterior diameters were nearly equal to the right and left diameters).

Tongue base hypertrophy was judged as lingual tonsillar hypertrophy and the hypertrophy of the tongue base oppresses the epiglottis and partially covers the glottis visible on awake laryngoscopy (25).

There were broadly six regions according to the examination procedure: nasal (nasal physical examination), oropharyngeal (oropharyngeal physical examination), jaw and neck physical examination, and RP and RG (evaluated by endoscopy). The presence of narrowing or obstruction was assessed at each region. The number of narrowing or obstruction regions was calculated (0–6 scores).

A regular overnight PSG (Compumedics, E-Series, Australia) monitoring was conducted. Six channels of electroencephalography signals (C3-M2, C4-M1, F3-M2, F4-M1, O1-M2, and O2-M1), two channels of electro-oculography signals (E1-M2 and E1-M2), chin electromyography (EMG) (EMG1-EMG2, EMG1-EMG3), electrocardiography, respiration (nasal pressure and airflow), oxygen saturation (SpO_2_), abdominal and chest movement, and leg movements were recorded according to the American Academy of Sleep Medicine (AASM) guidelines (26, 27). Sleep stage and respiratory events were analyzed manually by an experienced technician and reviewed by the registered polysomnographic technologist (RPSGT) (Registry Number: 18903) according to the AASM guidelines, version 2.3. The apnea-hypopnea index (AHI) (i.e., the sum of apnea and hypopnea events per hour) was calculated using the above monitoring indicators, according to the AASM manual (26, 27). Following the AASM guidelines, OSA was diagnosed and classified based on the AHI. Patients were classified into the two groups according to their AHI scores in the PSG as the mild OSA group (simple snorers and mild OSA, AHI <15) and the moderate-to-severe OSA (M-S OSA) group (AHI ≥15).

### Continuous Positive Airway Pressure Titration

Patients whose AHI was ≥15.0 per h and agreed to CPAP therapy were underwent full-night CPAP titration under PSG monitoring.

### Follow-Up

Patients who had pressure titration were followed-up by telephone in March 2021.

Questions regarding purchase of the CPAP, time of use per day, total time of use, improvement in clinical symptoms and comfort, and problems with ventilator use were asked in addition to whether the compliance was good (defined as over 4 h per day and more than 70% use per month).

### Statistical Analysis

Statistical analyses were performed using SPSS software version 23.0 (IBM SPSS Statistics, USA). Basic information, anatomical narrowing, morphology, and UA collapse were compared between the two groups. The normality of the distribution of the continuous variables was determined by the Shapiro–Wilk test. Normally distributed continuous variables are presented as mean ± SD. Nonnormally distributed continuous variables [age, body mass index (BMI), neck circumference (NC), and AHI] are presented as the median (25th−75th percentile) and analyzed using the Mann–Whitney *U* test. Categorical variables are presented as *n* (%) and compared using the chi-squared test. The Spearman's rank correlation coefficient was used for correlation analysis.

The multivariate logistic regression analysis was used to identify the independent risk factors associated with the risk of M-S OSA and initiation of home noninvasive ventilation from UA characteristics. First, a one-way analysis of the relevant factors was conducted and factors with *P* < 0.15 were included and those factors with significant collinearity were excluded. The backward method was used in the logistic regression model. *P* < 0.05 indicated statistical significance.

## Results

**Part 1, Comparisons of UA characteristics were made between patients with simple snorers and mild OSA and those with M-S OSA**.

### Characteristics of Baseline and UA Anatomical Narrowing

Of 1,186 patients, 184 patients without PSG data (*n* = 161), with incomplete laryngoscopic data (*n* = 19) and <18 years of age (*n* = 4) were excluded. Finally, 1,002 patients were included in the analysis (Figure 1). In the total sample, 827 and 175 patients were male and female, respectively. The mild OSA group included 276 patients, while the M-S OSA group included 726 patients.

There was no significant between-group difference in age [median (interquartile range): 45 years (35–55 years) vs. 46 years (37–55 years), *P* > 0.05]. The M-S OSA group had a significantly higher proportion of male patients (86.0 vs. 73.6%; *P* < 0.001) and significantly higher BMI [28.4 kg/m^2^ (25.8–31.1 kg/m^2^) vs. 25.9 kg/m^2^ (23.7–28.2 kg/m^2^)] and NC [41 cm (39–43 cm) vs. 39 cm (36–41 cm), *P* < 0.001] than the snorer-mild OSA group (Table 1).

**Table 1:** Distribution of upper airway narrowing by OSA severity.

	**Mild OSA group (AHI <15)**	**Moderate-to-severe OSA group** **(AHI ≥15)**	***P***
Age, years [median (IQR)]	45 (35–55)	46 (37–55)	0.168
BMI, kg/m^2^ [median (IQR)]	25.9 (23.7–28.2)	28.4 (25.9–31.1)	0.000
NC, cm [median (IQR)]	39 (36–41)	41 (39–43)	0.000
RP regions collapse during Müller maneuver [median (IQR)]	80% (64–100%)	90% (75–100%)	0.000
RG regions collapse during Müller maneuver [median (IQR)]	55% (50–75%)	55% (50–75%)	0.382
Gender (male:female)	203:73	624:102	0.000
Nasal septum deviation (%)	43.60%	45.10%	0.677
Nasal stenosis (%)	34.80%	32.50%	0.494
Nasal turbinate hypertrophy (%)	18.50%	24.20%	0.053
Thick and long uvulas (%)	39.90%	50.10%	0.004
Tonsillar hypertrophy (%)	2.30%	10.70%	0.000
Lateral pharyngeal wall stenosis (%)	7.70%	19.00%	0.000
Tongue hypertrophy (%)	42.70%	57.70%	0.000
Tongue base hypertrophy (%)	24.00%	34.60%	0.001
Friedman score ≥3 (%)	29.10%	33.70%	0.169
Mandibular retrusion (%)	41.20%	55.70%	0.000
Morphology of the retropalatal region (long oval and quasicircular) (%)	20.70%	39.00%	0.000
Positive Müller maneuver in the RP region (%)	72.50%	83.70%	0.000
Positive Müller maneuver in the RG region (%)	47.50%	53.30%	0.098
Positive Müller maneuver in both the RP and RG regions (%)	35.50%	44.50%	0.010

*A positive Muller maneuver in the RP region was defined as more than 75% of the collapsed area. A positive Müller maneuver in the RG region was defined as more than 50% of the collapsed area*.

*OSA, obstructive sleep apnea; AHI, apnea-hypopnea index; RP, retropalate; RG, retroglottal; BMI, body mass index; NC, neck circumstance*.

*Data were expressed as the median (25th−75th percentile) or the percentage*.

There were no significant between-group differences in the proportion of patients with nasal septum deviation (45.1 vs. 43.6%), presence of nasal stenosis on at least one side (32.5 vs. 34.8%), and presence of at least one nasal turbinate (24.2 vs. 18.5%) in the M-S OSA and the snorer-mild OSA groups (all *P* > 0.05). The M-S OSA group included a significantly higher proportion of patients with thicker and longer uvulas (50.1 vs. 39.9%, *P* < 0.05), patients with lingual hypertrophy (57.7 vs. 42.7%, *P* < 0.05), and patients with tonsillar hypertrophy (10.7 vs. 2.3%, *P* < 0.001). Furthermore, the proportion of patients with the Friedman score of ≥3 points was similar between the two groups (33.7 vs. 29.1%, *P* = 0.169). Meanwhile, the proportion of patients with lateral pharyngeal wall stenosis was significantly higher in the M-S OSA group (19.0 vs. 7.7%, *P* < 0.001) (Table 1).

### Morphology of the Retropalatal Region and Jaw Characteristics

The proportion of patients with a long oval and quasicircular shape retropalatal region (i.e., the anterior and posterior diameters were greater than or equal to the right and left diameters) was significantly higher in the M-S group than the snorer-mild OSA group (39.0 vs. 20.7%, *P* < 0.001). A long oval and quasicircular retropalatal region was associated with M-S OSA [odds ratio (OR): 2.45, 95% CI: 1.76–3.41, *P* < 0.001].

The proportion of patients with mandibular retrusion was higher in the M-S OSA group (55.7 vs. 41.2%, *P* < 0.05) (Table 1).

### Collapsibility of the RP and RG Regions

The rate of a positive Müller maneuver in the RP was significantly higher in the M-S OSA group than in the mild OSA group (83.7 vs. 72.5%, *P* < 0.001). The rate at the tongue base was also higher in the M-S OSA group than in the other group, but the difference did not reach statistical significance (53.3 vs. 47.5%, *P* = 0.098). The rate of a positive Müller maneuver in both the RP and RG regions was also significantly higher in the M-S OSA group (44.5 vs. 35.5%, *P* = 0.010) (Table 1).

The degree of collapse of the RP regions during Müller maneuver was significantly higher in the M-S OSA group than in the mild OSA group (*P* < 0.05). There were no significant differences in the degree of collapse of the RG regions between the two groups (*P* > 0.05).

### Regression Analysis

First, a one-way analysis of the relevant factors was conducted. The results of the univariate logistic regression analyses are shown in Table 2. Those factors with *P* < 0.15 were included and those with serious collinearity were excluded. Then, factors such as BMI, NC, sex, nasal turbinate hypertrophy, thick and long uvulas, tonsillar hypertrophy, narrowing of the lateral pharyngeal wall, tongue hypertrophy, tongue base hypertrophy, mandibular recession, morphology of the RP region, Müller maneuver in the RP, Müller maneuver in the RG, and degree of collapse of the RP regions during Müller maneuver were brought into the regression model. The backward method was used in the regression model. Finally, factors such as BMI, NC, tongue base hypertrophy, tonsillar hypertrophy, and mandibular recession were entered into the final regression model, which were indicated as independent predictors for M-S OSA (Table 2).

**Table 2:** Association between the characteristics of the upper airway structure and severity of OSA in the regression model.

**Dependent variable**	**Independent variable**	**B**	***P***	**OR (95% CI)**
Moderate	BMI	0.126	0.000	1.134 (1.061–1.213)
and severe	Neck circumstance	0.105	0.004	1.111 (1.035–1.192)
OSA	Tonsillar hypertrophy	1.984	0.007	7.274 (1.708–30.976)
	Tongue base hypertrophy	0.617	0.005	1.854 (1.204–2.856)
	Mandibular recession	0.693	0.000	2.000 (1.397–2.866)

*OSA, obstructive sleep apnea; BMI, body mass index; OR, odds ratio; RG, retroglottal; RP, retropalate; CI, confidence interval*.

**Part 2, to explore the effect of UA characteristics on CPAP titration pressure and initiation and compliance of CPAP therapy**.

### Association Between the UA Characteristics and CPAP Titration

Of 1,002 patients enrolled above, 138 patients underwent noninvasive ventilation pressure titration under PSG monitoring in a sleep lab. Of these patients, bilevel positive airway pressure (BiPAP) titration was applied in 19 patients and CPAP titration was performed in 119 patients. The relationship between UA characteristics and CPAP titration was analyzed in 119 patients who underwent CPAP application.

Compared to the control subjects, there were no significant differences in CPAP titration among the groups of deviated septum, nasal stenosis, nasal turbinate hypertrophy, tongue base hypertrophy, mandibular retrusion, Friedman score ≥3, morphology of the retropalatal region as long oval and quasicircular, and positive Müller maneuver in the RP or RG regions (*P* > 0.05).

The CPAP titration pressure of men was higher than that of women (12.1 ± 2.2 vs. 10.7 ± 2.0 cm H_2_O, *P* = 0.038). The CPAP values in patients with thick and long uvulas, tonsillar hypertrophy, lateral pharyngeal wall stenosis, and tongue hypertrophy were higher than those in subjects of the control group (12.5 ± 2.2 vs. 11.2 ± 2.1, 13.2 ± 2.0 vs. 11.8 ± 2.3, 12.7 ± 2.3 vs. 11.6 ± 2.1, 12.4 ± 2.3 vs. 11.5 ± 2.1, respectively, *P* < 0.05).

There was a positive correlation between CPAP and BMI and NC (*r* = 0.389, 0.410, respectively, *P* = 0.000) and a weak negative correlation with age (*r* = −0.297, *P* = 0.001).

### Effect of UA Characteristics on the Initiation of Home CPAP Therapy and 3-Month Adherence

Of 119 patients who received CPAP titration, 93 patients were followed-up by telephone.

There were no significant differences of gender, age, BMI, NC, deviated nasal septum, nasal stenosis, morphology of the RP region, tongue base hypertrophy, Friedman score of ≥3, thick and long uvula, tonsillar hypertrophy, mandibular retrusion, lateral pharyngeal wall stenosis, and tongue hypertrophy were not significantly different between the initiation and not-initiation home CPAP therapy groups (*P* > 0.05) (Table 3).

**Table 3:** Effect of upper airway (UA) characteristics on the initiation of home continuous positive airway pressure (CPAP) therapy.

	**Not initiation of home CPAP group**	**Initiation of home CPAP group**	***P***
Gender (male: female)	5: 33	7: 48	0.951
Age, years [median (IQR)]	43.5 (36.0, 57.3)	47.0 (36.0, 59.0)	0.409
BMI, kg/m^2^ [median (IQR)]	28.4 (26.0, 31.9)	29.1 (26.6, 32.5)	0.501
NC, cm [median (IQR)]	41.0 (40.0, 43.8)	42.0 (40.0, 44.0)	0.882
Nasal septum deviation (%)	34.2%	22.6%	0.223
Nasal stenosis (%)	42.1%	32.7%	0.356
Nasal turbinate hypertrophy (%)	31.6%	12.7%	0.027
Morphology of the retropalatal region (long oval and quasicircular) (%)	36.8%	36.4%	0.962
Positive Müller maneuver in the RP region (%)	71.1%	90.7%	0.014
Tongue base hypertrophy (%)	13.2%	21.8%	0.288
Friedman score ≥3 (%)	21.1%	29.1%	0.384
Thick and long uvulas (%)	52.6%	52.7%	0.993
Tonsillar hypertrophy (%)	10.8%	16.3%	0.465
Mandibular retrusion (%)	55.3%	38.2%	0.104
Lateral pharyngeal wall stenosis (%)	17.1%	25.0%	0.384
Positive Müller maneuver in the RG region (%)	39.5%	63.6%	0.022
Positive Müller maneuver in both the RP and RG regions (%)	31.6%	58.2%	0.012
Tongue hypertrophy (%)	65.8%	56.4%	0.361

*Data were expressed as the actual value, median (25th−75th percentile) or the percentage*.

There were significant differences between the two groups for nasal turbinate hypertrophy and positive Müller maneuver in the RP, RG, and both the regions (*P* < 0.05) (Table 3).

The multivariate logistic regression analysis was applied with the backward method. Variables with *P* < 0.15 in the univariate logistic regression analysis were included and those with severe covariance were excluded. The final variables included in the regression equation were nasal turbinate hypertrophy, mandibular retrusion, and positive Müller maneuver in the RP and RG regions (Table 4).

**Table 4:** Association between UA characteristics and the initiation of home CPAP treatment in the regression model.

**Dependent variable**	**Independent variable**	**B**	***P***	**OR (95% CI)**
Initiation of home CPAP	Nasal turbinate hypertrophy	−1.358	0.021	0.257 (0.081–0.813)
	Mandibular retrusion	−1.143	0.027	0.319 (0.116–0.876)
	Positive Müller maneuver in the RP region	1.637	0.014	5.140 (1.393–18.962)
	Positive Müller maneuver in the RG region	1.251	0.012	3.495 (1.309–9.327)

*OR, odds ratio; CI, confidence interval; CPAP, continuous positive airway pressure; UA, upper airway*.

Of 93 patients who received home CPAP therapy, 18 patients had <3 months of home CPAP therapy or had poor compliance and 55 patients had more than 3 months of home CPAP therapy and had good compliance.

The univariate logistic regression analysis results showed that the group with good compliance for 3 months had a higher proportion of morphology of the retropalatal region as long oval and quasicircular and a higher proportion of thick and long uvula than those of the control group (45.9 vs. 16.7%, 62.2 vs. 33.3%, respectively, *p* < 0.05).

## Discussion

This study shows that the characteristics of the UA influence the severity of OSA, CPAP pressure, and home CPAP therapy. Patients with M-S OSA had multiple sites of velopharyngeal and glossopharyngeal narrowing and increased functional collapse. Tongue root hypertrophy, tonsillar hypertrophy, mandibular recession, NC, and BMI were independent predictors for M-S OSA. The CPAP pressures in patients with thick and long uvulas, tonsillar hypertrophy, lateral pharyngeal wall stenosis, and tongue hypertrophy were higher than those of the control group (*P* < 0.05, respectively). Nasal turbinate hypertrophy, mandibular retrusion, and positive Müller maneuver in the RP and RG regions were independent predictors for the initiation of home CPAP treatment.

Although the role of neuroregulation in the development of OSA has been emphasized in the recent years (28, 29), UA anatomy and function are still important factors affecting OSA. In this study, the M-S OSA group had a higher proportion of patients with a long uvula, tongue hypertrophy, hypertrophy of the tongue root, narrowing of the lateral pharyngeal wall, tonsillar hypertrophy, and Friedman scores ≥3. The M-S OSA group included a higher proportion of patients with a positive Müller maneuver in the RP region and tended to have a shortened left-right diameter of the RP region. It is unclear whether patients with flat oval and quasicircular shapes are more prone to snoring or whether the change in pharyngeal morphology is due to the spasm of the UA muscles caused by sleep apnea. Anyway, stenosis of the UA anatomy might be an important factor causing severe OSA in the Chinese population. In addition, this study showed that the BMI of patients with M-S OSA was only 28.4 kg/m^2^, which is lower than that of the OSA population reported in the West (30, 31). It suggested that even if patients with OSA in China are not overweight, the severity of OSA may be high. This may be related to the multiplane and multisite stenosis.

For the characteristics of the UA, in addition to the narrowing of the anatomical structures, there is functional collapse. UA collapsibility plays a crucial role in the physiology of UA. This study showed that there was a higher proportion of Müller maneuver positivity in the RP in the M-S OSA group than in the mild OSA group, suggesting an increased collapsibility. The collapse pattern and degrees can be assessed directly by Müller maneuver under awake endoscopy or DISE. Previous studies have showed that the aging effect on OSA collapse has been demonstrated to be associated with OSA severity (32, 33). Some studies showed that older age correlated with multisites obstruction, including palatopharyngeal and hypopharyngeal level obstructions (32). Other studies showed that elderly patients showed a higher incidence of total collapse in the velum region compared to younger patients and no difference in tongue base collapses (33).

The musculature of the pharynx has complex and sophisticated functions, which is not only related to respiration, but also to swallowing function. Studies reported that there were pharyngeal swallowing impairments in patients with OSAs, including delayed initiation of pharyngeal swallow and penetration/aspiration (34). Olfactory function has also been correlated with the presence of OSA disorders, particularly with more symptoms in patients with more severe apneas (35, 36). Olfaction is one of the five neurosensory systems and cognitive impairment can influence on olfactory function. When considering the negative effects of OSA on cognitive functions, patients with OSA should be warned against olfactory function decline (36).

The evaluation of the UA is mainly used in the surgical setting. Few studies have examined the role of endoscopy in internal medicine. This study revealed the anatomical characteristics of the UA in patients with OSA who visited an internal medicine department. Since changes in the UA are the main causes of OSA, assessing the UA is necessary for the management of OSA. The determination of UA anomalies is associated with the titration of CPAP in the internal medicine department (14).

This study showed that the CPAP titration pressures were associated with thick and long uvulas, tonsillar hypertrophy, lateral pharyngeal wall stenosis, and tongue hypertrophy (*P* < 0.05, respectively). Hypertrophy of the soft tissues of the pharynx causes narrowing of the airway, which exacerbates the increased airway resistance in the presence of decreased UA muscle tone during the sleep state. Greater pressure support is required to open the UA. Therefore, it is a key factor affecting the pressure of CPAP therapy. A study assessed the UA features in patients with OSA by CT during Müller maneuver and the results showed that BMI and the UA length were independently associated with CPAP titration pressure (*p* < 0.05) (14).

Second, the characteristics of the UA were also found to influence the acceptance and compliance with home CPAP therapy in this study. Nasal turbinate hypertrophy, mandibular retrusion, and the collapse of UA represented by Müller maneuver were independent predictors for the initiation of home CPAP treatment (Table 4). Among them, nasal turbinate hypertrophy and mandibular retrusion had a negative effect on the initiation of home ventilator therapy.

The assessment of the respiratory airways must also include the assessment of nasal function. Although nasal septal deviation or turbinal hypertrophy has not been shown to represent a site of UA collapse, nasal surgery has been shown to improve both the CPAP compliance and snoring severity (37, 38).

From a clinical point of view, nasal problems are indeed an important factor affecting patients receiving ventilator therapy. Previous studies have shown similar results that higher grades of hypertrophic change of the inferior turbinate were observed more in the CPAP nonadherent group (15). Nasal resistance was higher in patients who discontinued CPAP therapy and nasal disease and nasal parameters are key factors for early CPAP therapy discontinuation (39). The excessive UA blockage in the nasal cavity might cause the subjective discomfort that decrease CPAP adherence.

On the other hand, the collapsibility of UA was a positive factor influencing home CPAP therapy, indicating that patients with high airway collapsibility were prone to accept home CPAP therapy. The collapse of UA represented by Müller maneuver is more suggestive of functional collapse, which might be more suitable for CPAP therapy. In those patients who had initiated home CPAP therapy, follow-up was performed to assess adherence at 3 months. Results revealed that UA characteristics including morphology of the retropalatal region and thick and long uvula were also associated with 3-month adherence. The exact mechanism is still unclear. According to the results of the previous part of this study, the RP region tends to have a flat oval shape in mild patients with OSA, while it tended to have an elongated oval and quasicircular shape in patients with M-S OSA. The change in pharyngeal morphology might be due to the spasm of the UA muscles caused by sleep apnea and, thus, sustained CPAP treatment has the potential to restore the normal morphology of the RP region.

For internists, the evaluation of UA characteristics may have significance for OSA precision treatment, classification, prediction of CPAP treatment pressure, and treatment compliance (14, 20).

Transnasal endoscopy is an important technique for the visualization of the anatomical narrowing of the UA and the assessment of its functional status. This technique can be applied in three ways. DISE is currently the most important and the forefront method in evaluating the UA obstruction (40, 41). It involves sleep induction using anesthetic drugs and evaluating the UA with an endoscope (10, 33). It is of great significance to evaluate the site and degree of collapse to select the surgical method and predict the surgical efficacy before ENT surgery (42). However, this approach has its limitations. For example, there are differences between narcotics-induced sleep and real sleep as well as differences in the sleep state induced by different drugs and the depth of sedation (43). Moreover, there are certain risks in using anesthetic drugs to induce sleep (12, 41). In patients with OSA, the UA is prone to stenosis and collapse, aggravating during drug-induced sleep, thus requiring careful monitoring (8). Some studies suggest that the most severe obstruction seen on DISE is not necessarily the narrowest, but may simply be the weakest site. Therefore, not all the cases are suitable for a relatively complex evaluation such as the DISE. Natural sleep endoscopy can show the structure of the UA during natural sleep, which has the advantage of displaying the actual patient's condition without the interference of drugs (11, 12). However, natural sleep endoscopy is time-consuming and laborious, since it requires patients to fall asleep by themselves, which might not be easy and there is a risk of waking the patient when inserting the endoscope. Awake endoscopy is advantageous in a comprehensive sleep center, considering its simplicity, convenience, and safety. It does not require special care or monitoring, since drugs are not used. Using our UA assessment system assisted by endoscopy, we observed only a few nosebleeds and no significant adverse effects or intolerance. The main disadvantage of awake endoscopy is that the physiology is inconsistent with sleep when OSA occurs. Some previous studies compared waking laryngoscope to drug-induced endoscopy (40, 44, 45). Still, the velum and oropharynx positions showed good correlations between the waking state and drug-induced sleep. However, at the level of the tongue base, the correlations of both the assessments are uncertain (44, 46). Therefore, this study suggests that UA evaluation in waking state is a safe and effective method for OSA UA evaluation in sleep centers dominated by internal medicine.

This study has limitations. This was a retrospective cross-sectional study and, thus, the causal relationship of UA anatomical stenoses and functional collapse with OSA could not be clarified. Moreover, this study was performed in a population undergoing laryngoscopy for assessment of the UA rather than in the general population; as such, the possibility of selection bias could not be eliminated. The CPAP therapy follow-up is a telephone follow-up rather than an outpatient follow-up and may have inaccurate or incomplete information. More information would be available, if SD data from the patient's CPAP could be downloaded and analyzed.

In conclusion, patients with M-S OSA have multiple loci of velopharyngeal and glossopharyngeal stenosis and collapse. The UA characteristics also affect CPAP titration pressure and compliance. Awake state assessment of UA (UA physical examination, laryngoscopy, and Müller maneuver) is a safe, convenient, and effective assessment method in internal medicine.

Therefore, we recommend that endoscopy-assisted UA examination can be used in sleep centers dominated by internal medicine where available. This allows for a more precise assessment of the indications for patient treatment. Refer appropriate patients to the appropriate specialty for specialist care. This is formally in line with the principles of multidisciplinary care. Most patients are first seen in internal medicine and this system of assessment is more conducive to multidisciplinary cooperation, improved management of OSA and help precision medical practice (47).

## Data Availability Statement

The raw data supporting the conclusions of this article will be made available by the authors, without undue reservation.

## Ethics Statement

This study was approved by the Ethics Committee of Peking University (2020-562). Written informed consent for participation was not required for this study in accordance with the national legislation and the institutional requirements.

## Author Contributions

CZ is responsible for data sorting, statistical analysis, and the writing. GW participated in research design, conception, and planning. JM is responsible for the research design, data analysis, and article modification. YS and YG are respectively responsible for the input and sorting of PSG and laryngoscope data. MC is responsible for data entry. All authors contributed to the article and approved the submitted version.

## Funding

This study was supported by the Foundation of National Natural Science (Grant Number 82000096).

## Conflict of Interest

The authors declare that the research was conducted in the absence of any commercial or financial relationships that could be construed as a potential conflict of interest.

## Publisher's Note

All claims expressed in this article are solely those of the authors and do not necessarily represent those of their affiliated organizations, or those of the publisher, the editors and the reviewers. Any product that may be evaluated in this article, or claim that may be made by its manufacturer, is not guaranteed or endorsed by the publisher.
